# Initiation and termination of DNA replication during S phase in relation to cyclins D1, E and A, p21^WAF1^, Cdt1 and the p12 subunit of DNA polymerase δ revealed in individual cells by cytometry

**DOI:** 10.18632/oncotarget.4149

**Published:** 2015-05-15

**Authors:** Zbigniew Darzynkiewicz, Hong Zhao, Sufang Zhang, Y.W.T. Lee Marietta, Y.C. Lee Ernest, Zhongtao Zhang

**Affiliations:** ^1^ Brander Cancer Research Institute, Department of Pathology, New York Medical College, Valhalla, NY; ^2^ Department of Biochemistry and Molecular Biology, New York Medical College, Valhalla, NY

**Keywords:** cell cycle, S-phase, cell proliferation, laser scanning cytometry, DNA replication, EdU labeling, polymerase δ

## Abstract

During our recent studies on mechanism of the regulation of human DNA polymerase δ in preparation for DNA replication or repair, multiparameter imaging cytometry as exemplified by laser scanning cytometry (LSC) has been used to assess changes in expression of the following nuclear proteins associated with initiation of DNA replication: cyclin A, PCNA, Ki-67, p21^WAF1^, DNA replication factor Cdt1 and the smallest subunit of DNA polymerase δ, p12. In the present review, rather than focusing on Pol δ, we emphasize the application of LSC in these studies and outline possibilities offered by the concurrent differential analysis of DNA replication in conjunction with expression of the nuclear proteins. A more extensive analysis of the data on a correlation between rates of EdU incorporation, likely reporting DNA replication, and expression of these proteins, is presently provided. New data, specifically on the expression of cyclin D1 and cyclin E with respect to EdU incorporation as well as on a relationship between expression of cyclin A vs. p21^WAF1^ and Ki-67 vs. Cdt1, are also reported. Of particular interest is the observation that this approach makes it possible to assess the temporal sequence of degradation of cyclin D1, p21^WAF1^, Cdt1 and p12, each with respect to initiation of DNA replication and with respect to each other. Also the sequence or reappearance of these proteins in G_2_ after termination of DNA replication is assessed. The reviewed data provide a more comprehensive presentation of potential markers, whose presence or absence marks the DNA replicating cells. Discussed is also usefulness of these markers as indicators of proliferative activity in cancer tissues that may bear information on tumor progression and have a prognostic value.

## INTRODUCTION

Multiparameter cytometry combined with gating analysis offers the means to correlate expression of different cellular entities and events with each other in individual cells. Numerous attempts have been made to use this methodology to assess a relationship between DNA replication and expression of particular nuclear proteins in order to explore their role in this critical event of the cell cycle. During the past four decades the most widely used cytometric methodology to detect DNA replication was based on immunocytochemical detection of 5-bromo-2-deoxyuridine (BrdU) [1]. However, because this methodology requires denaturation of DNA either by heat or strong acid to make the incorporated BrdU accessible to BrdU-Ab, it was incompatible with a concurrent immunocytochemical detection of nuclear proteins whose epitopes were damaged under these harsh conditions. The subsequent approaches to detect DNA replication that were compatible with simultaneous detection of protein, such as partial digestion of DNA with restriction nucleases or endonuclease [2], or DNA strand break induction by photolysis (SBIP) [3] had other limitations that prevented their widespread use. The recently introduced methodology of labeling DNA with 5-ethynyl-2′-deoxyuridine (EdU) which is detected with fluorochrome-tagged azides by a copper (I) catalyzed [3+2] cycloaddition reaction, defined as “click chemistry”, has no such limitations. EdU therefore has become now the preferred DNA precursor applicable in flow and imaging cytometry [4-7]. It should be noted however, that the application of EdU in experiments that require long-term incubation following the labeling has constrains because the incorporated EdU causes perturbation of the cell cycle progression, DNA damage and cytotoxicity [8-10].

In our prior studies we correlated DNA replication as detected by EdU incorporation with the expression of γH2AX and ATM in response to DNA damage induced by oxidative stress [11, 12], DNA topoisomerase I inhibitors camptothecin or topotecan, topoisomerase II inhibitors mitoxantrone or etoposide [13] as well as UV irradiation [14]. Of particular interest were our findings that genotoxic agents camptothecin, topotecan and UV irradiation induced DNA damage selectively in DNA replicating cells, and upon examination by confocal microscopy, at the sites of DNA replication foci. In contrast, in the cells subjected to oxidative stress or treated with DNA topoisomerase II inhibitors, the DNA damage response was induced in the cells regardless of their DNA replication status, and in the case of DNA replicating cells also outside of DNA replication foci [11-14]. We have also reported that assessment of the frequency of cells initiating and terminating DNA replication during the pulse exposure to EdU offers information on the kinetics of initiation and termination of DNA replication at the onset and end of the S phase, respectively [15]. Recent studies by Furia et al., [16-19] utilizing a novel imaging computational platform named Automated Microscopy for Image Cytometry (AMICO) provided an excellent example of analysis of a correlation of DNA replication as revealed by EdU incorporation and expression of proteins such as cyclin A, cyclin E, Ki-67, p53, and 53BP, the latter detected either integrated over the nucleus or located within the 53BP nuclear foci. Using mammary epithelial cells these authors were able to critically evaluate the correlation between different stages of DNA replication (early-S, mid-S, late-S) and expression of these proteins [16-19].

In recent studies we applied similar approaches to assess the expression of several nuclear proteins associated with initiation and termination of DNA replication that made it possible to reveal the changeable kinetics of their expression at the early and late sections of the S phase [20]. The report was focused on mechanisms associated with a role of the detected proteins with DNA replication, particularly in relation to a function of DNA polymerase δ (Pol δ). The multiparameter imaging - laser scanning cytometry (LSC) [21] served in these experiments as a tool to explore these mechanisms. In the present review rather than focus on control of Pol δ by p12 degradation we emphasize the application of imaging cytometry and outline possibilities offered by the concurrent differential analysis of DNA replication in conjunction with expression of the nuclear proteins. Also a more extensive analysis of the data reporting a correlation (or lack thereof) between the rate of DNA replication (EdU incorporation) and expression of these proteins, is presently provided. Our findings can be compared with the mentioned data of Furia at al., [15-19] who, as mentioned, used a somewhat different methodological approach to assess the association between DNA replication and expression of some of these proteins.

Using the prior described methodology [11-15, 20] we also report new data, specifically these related to expression of cyclin D1 and cyclin E with respect to EdU incorporation. Moreover, we demonstrate the differential staining of the DNA replication-associated proteins (cyclin A, Ki-67) concurrently with the proteins that are degraded prior to initiation of DNA replication (p21, Cdt1), in the same individual cells. The reviewed data further underscore the analytical capability of multiparameter cytometry in exploring factors associated with initiation or termination of DNA replication and provide a more comprehensive presentation of potential markers, whose presence or absence marks the DNA replicating cells. Discussed is also usefulness of these markers as indicators of proliferative activity in cancer tissues that may bear information on tumor progression and have a prognostic value in pathology.

## NEGATIVE MARKERS OF DNA REPLICATION: CYCLIN D1, THE CDK INHIBITOR P21^WAF1^, THE DNA REPLICATION LICENSING FACTOR Cdt1 AND p12, THE SMALLEST SUBUNIT OF DNA POLYMERASE Δ

### Cyclin D1

Cyclins are the key constituents of the cell cycle progression machinery. Combined with their respective cyclin-dependent serine/threonine protein kinases (CDKs) they form the holoenzymes that phosphorylate different sets of target proteins thereby driving the cell through consecutive phases of the cycle. Their kinase activity is additionally modulated by Cdk inhibitors (CKIs), and close cooperation between cyclins, CDKs and CKIs is necessary for ensuring the ordered progression through the cell cycle [reviewed in 22-27]. It should be noted, however, that in addition to their well-characterized function in cell cycle control, mammalian cyclins, CDKs and CKIs are also involved in processes such as transcription, epigenetic regulation, metabolism, stem cell self-renewal, neuronal functions and spermatogenesis [22].

The cellular abundance of cyclin D1, as of other cyclins, is regulated by their synthesis and degradation. The synthesis of cyclin D1 is induced by growth factors that stimulate the MAPK/ERK (Ras-Raf-MEK-ERK) pathways [23]. The MAPK member of these pathways activates a transcription factor Myc which then activates the critical positive cell cycle regulators that include Cdks, cyclins and E2F transcription factors [24, 25]. Degradation of cyclins takes place through an anaphase-promoting complex/cyclosome (APC/C) dependent pathway [26, 27]. The APC/C is an E3 ubiquitin ligase that targets specific substrates for degradation by the 26S proteasome [27]. Cyclin D1 interacts with Cdk2, Cdk4 and Cdk6. The cyclin D-Cdk4/6 complex partially phosphorylates retinoblastoma tumor suppressor protein (pRb) which is the key event facilitating cell progression through G_1_, up to the entrance to S phase. Specifically, partial phosphorylation of pRb by the cyclin D-Cdk4/6 kinase releases E2F factors that activate transcription of several genes, including that of cyclin E, that being subsequent to cyclin D1, promotes the entrance to S [28, 29].

**Figure 1:**
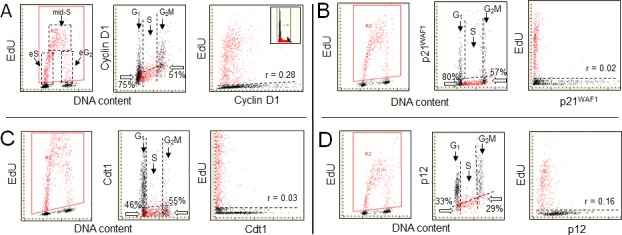
Expression of cyclin D1 (A), the CDK inhibitor p21 (B), the chromatin licensing and DNA replication factor Cdt1 (C), and the smallest subunit of DNA polymerase δ p12 (D), in relation to EdU incorporation A549 cells were exposed to EdU for 60 min, the EdU incorporation was detected by the Click-It^TM^ protocol, cellular expression of cyclin D1, p21, Cdt1 and p12 was detected immunocytochemically, DNA was counterstained with DAPI and cellular fluorescence was measured by LSC, as described [11-15,20]. During the “*paint-a-gate*” analysis the cells incorporating EdU were colored red. The dashed contours in A (left panel) outline the cells that during duration of the 60 min exposure to EdU were entering S (eS), were constantly present during pulse duration (mid-S), or were entering G_2_ (eG_2_) [15]. The EdU incorporation is correlated on the bivariate scatterplots with expression of these proteins presented as integrated values of immunofluorescence intensity over cell nucleus (mid-panels). The dashed skewed lines in mid-panels show the upper level of fluorescence intensity of the cells stained with the secondary fluorochrome-tagged Ab only, namely +3SD of the mean fluorescence intensity of such negative control [20]. The cells below this line are thus considered negative with respect to expression of these proteins. Below the thick arrows in mid-panels presented is the percentage of cells at the G_1_ to S transition (with DNA content equivalent of G_1_) and at the S to G_2_ transition (G_2_M DNA content) that are negative with respect to expression of the measured proteins and *did not incorporate EdU*. A direct relationship between expression of these proteins and EdU incorporation is presented in the right panels in which the dashed line separates the EdU labeled from unlabeled cells. The regression analysis reveals the degree of correlation (Pearson; r = x) between incorporation of EdU and expression of each of these proteins in all EdU positive cells, assessed as described before [20].

Figure 1 (A) illustrates the relationship between EdU incorporation and expression of cyclin D1. The cell cycle specific expression of cyclin D1, as shown in this figure (mid-panel) is consistent with that as initially defined by flow cytometry [30, 31]. The data show that a majority of cells (identified by their DNA content as in the S-phase and incorporating EdU) essentially were cyclin D1 negative. Among the cyclin D1 negative cells with DNA content equivalent of G_1_ 75% cells did not initiate DNA replication during exposure to the precursor. Thus, the remaining 25% of the cyclin D1 negative/EdU positive cells with a G_1_ DNA content, *were entering S* phase during this time (eS). However, they still are identifiable, based on intensity of DAPI fluorescence (DNA content), as in G_1_ because their DNA content during that period increased so minimally that they cannot be distinguished from the genuine G_1_ cells. The presence of a predominant proportion of cyclin D1 negative cells not yet incorporating EdU indicates that near complete degradation of this protein had to occur quite ahead to initiation of EdU incorporation during the transition from G_1_ to S.

At the S to G_2_ transition the cohort of cells exposed to the precursor during duration of the EdU pulse entered G_2_ and were identified as the EdU-positive G_2_M cells. Because there were 51% cyclin D1 negative-EdU unlabeled cells, the synthesis and accumulation of cyclin D1 has to take place at a certain time following termination of DNA replication. However, the bivariate cyclin D1 *vs* EdU scatterplot (right panel) shows a relatively weak correlation (Pearson; r = 0.28) between incorporation of EdU and expression of cyclin D1. This correlation apparently stems from the fact that the EdU labeled cells entering G_2_ during the duration of the pulse initiate the synthesis of cyclin D1. Thus, it is likely that the re-expression of cyclin D1 in G_2_, although it starts after termination of EdU incorporation, has an onset of synthesis in less than 60 min (duration of the EdU pulse) following the end of EdU incorporation (S to G_2_ transition). As described further in the review, the immunocytochemical detection of proteins suffers certain shortcomings that should be taken into an account when analyzing this type of data.

We have recently utilized the EdU-labeling method to analyze the degradation of three proteins, p21^WAF1^, Cdt1 and the p12 subunit of DNA polymerase δ (Pol δ) [20]. Here, we review these findings, as they relate to the correspondence of their degradation at the onset of DNA synthesis and their reappearance during G_2_/M. Also of note, we wish to illustrate the insights that can be gained by multi-parametric analysis offered by LSC in combination with the identification of replicating cells by EdU pulse-labeling. Moreover, the p21^WAF1^, Cdt1 and p12 are linked by a common mechanism for their degradation by CRL4^Cdt2^, which regulates the G_1_/S transition and the licensing of replication origins by the loading of the MCM proteins [32, 33].

### p21^WAF1^

The protein p21^WAF1^ is a cyclin-dependent kinase inhibitor (CKI) which binds and inhibits the activity of cyclin-CDK2, -CDK1, and -CDK4/6 complexes, and thus functions as a checkpoint regulator of cell cycle progression at G_1_ and S phase [34-37]. The expression of this gene is induced by the tumor suppressor p53 in response to a variety of stimuli, particularly from DNA damage [36]. In addition to cell arrest in G_1_, the expression of this protein can mediate cellular senescence [38, 39]. However, p21^WAF1^ levels are also regulated by posttranslational means, via its degradation by E3 ubiquitin ligases, and has multiple cellular functions during the normal cell cycle, largely mediated by its high affinity for PCNA [32, 33]. Its degradation during G_1_/S, in concert with those of Cdt1 and the histone methylase Set8, play key roles in the prevention of re-replication [40, 41].

Figure 1B illustrates the relationship between EdU incorporation and p21 levels. Similar as in the case of cyclin D1, the absence of p21 characterizes the EdU incorporating cells, revealing that degradation of p21 occurs prior to initiation of DNA replication. The frequency of cells with a G_1_ DNA content that are both p21- and EdU- negative (80%) is similar to that of cyclin D1- and EdU- negative cells (A, 75%). The closeness of these numbers indicates that prior to initiation of DNA replication degradation of cyclin D1 as well as of p21 occurs at approximately the same time. Likewise, the percent of cells with a G_2_M DNA content that are both p21 and EdU- negative (57%) is similar to that of cyclin D1- and- EdU negative (A; 51%). Thus, following termination of DNA replication, the accumulation of p21 as well as of cyclin D1, occurs at approximately the same time. Among the EdU positive cells no correlation between expression of p21 and EdU incorporation is apparent (r = 0.02). Seen from another perspective, the cellular regulation of p21 is such that its levels are reduced prior to the onset of DNA replication.

### DNA replication factor Cdt1

The Cdc10-dependent transcript 1 (Cdt1) is essential for formation of the pre-replicative complexes (preRC) and is a key licensing factor that binds to the origin recognition complex, and together with Cdc6 loads the MCM2-7 (minichromosome maintenance subunits 2-7), and licenses the complex for replication [32, 33, 42-44]. Its low level/absence during S phase is a consequence of its targeted destruction during the G_1_/S transition that is required to prevent re-replication. The master regulator of Cdt1 is CRL4^Cdt2^, which orchestrates the degradation of Cdt1, p21, Set8 (histonemethylase Set8) [32, 33] as well as p12 [45, 46], via their possession of a common peptide motif, the PIP-degron. The PIP-degrons as a group have higher affinities for PCNA than PIP-boxes [41], and thus Cdt1, in addition to its functions as a licensing factor, may also have inhibitory effects on PCNA binding processes, as reported recently for the translesion polymerases Pol η and Pol κ [48]. This property is similar to that attributed to p21^WAF1^ [32, 33].

The relationship between the expression of Cdt1 and DNA replication is shown in Figure 1C. As in the case of cyclin D1 (Figure 1A) and p21 (Figure 1B) the cells incorporating EdU appear to be devoid of Cdt1. Here, the analysis of the cells in the G_1_/S transition region shows that among the Cdt1-negative cells 46% of them were not incorporating EdU. Thus, the time-lapse between degradation of Cdt1 and initiation of EdU incorporation appears to be somewhat shorter compared to p21 or cyclin D1. As mentioned the licensing factor Cdt1 binds to the origin recognition complex, together with Cdc6 loads the MCM2-7, and licenses the DNA within the complex for replication. Its presence at the end of G_1_, just prior to initiation of DNA replication is required. The data shown in Figure 1C are consistent with the notion that initiation of DNA replication is concurrent with degradation of this protein to the level that cannot be immunocytochemically detected. Relatively fewer Cdt1-positive cells are present in G_2_M compared with the cyclin D1, p21-, or p12- positive cells (Figure 1D). This indicates that unlike in the case of cyclin D1-, p21 or p12 the accumulation of Cdt1, while is detected in some cells during G_2_M, in most cells takes place after the cell division, in G_1_ phase of the cell cycle.

### p12, the smallest subunit of DNA polymerase δ

DNA polymerase δ (Pol δ) together with Pol ε are the primary polymerases responsible for DNA replication in eukaryotes. Human Pol δ consists of four subunits: p125, p68, p50 and p12 [49-51]. In response to DNA damage p12 undergoes degradation which results in conversion of Pol δ4 to Pol δ3, the trimer lacking p12 [45, 53, 54]. Thus far, two E3 ligases RNF8 and CRL4^Cdt2^ have been identified to participate in targeting p12 for degradation after DNA damage [46, 55]. While initially Pol δ3 was shown to be implicated in DNA repair, subsequent studies indicated that it is the main form of Pol δ that is involved in DNA replication as well [20, 45, 56, 57]. Consistent with this are the observations on cells synchronized in the cell cycle that Pol δ3 is being formed in S-phase cells [45]. We have recently reported that with no synchronization, under conditions of unperturbed growth, during progression through the cell cycle DNA replication occurs in the cells in which p12 levels have fallen to near-baseline levels, at least as judged within the sensitivity of the antibody used for its immunocytochemical detection [20, 45]. The functions of p12 are complex and some appear to be related to maintenance of genome integrity as demonstrated by the findings that depletion of p12 leads to genomic instability [58, 59].

Figure 1D illustrates the actual relationship between the expression of p12 and DNA replication. The bivariate distributions presenting the cells expressing p12 vis-à-vis EdU incorporation resemble those of cyclin D1 (Figure 1A) p21^WAF1^ (Figure 1B) and Cdt1 (Figure 1C). Specifically, they show absence of the respective proteins in the cells incorporating EdU. There are distinctly fewer cells with a G_1_ DNA content that are p12 negative and not incorporating EdU (33%) than of the cyclin D1- (75%) or p21- (80%) negative cells, respectively. This indicates that the initiation of DNA replication occurs earlier after the loss of p12 compared with the loss of p21 or cyclin D. In other words, the “time window” between the degradation of p12 and onset of DNA replication is shorter compared to that of the degradation of cyclin D or p21 and thus degradation of these proteins precedes that of p12 degradation. Also, fewer p12 negative cells with a G_2_M DNA content did not incorporate EdU (29%) than did the cyclin D1 (51%)- or p21 (57%)- negative cells. These data in turn indicate that following termination of DNA replication the accumulation of p12 is detected prior to the accumulation of cyclin D1 or p21. Similar to p21 no evidence is apparent on a correlation among the EdU positive cells between expression of p12 and EdU incorporation (r = 0.16).

## KINETIC INFORMATION ON A SEQUENCE OF DEGRADATION OF CYCLIN D, P21^WAF1^, CDT1 AND P12 WITH RESPECT TO INITIATION OF DNA REPLICATION

Most information on a sequence of alterations of the levels of a particular protein with respect to DNA replication is generally obtained by Western blotting combined with cell synchronization. Such approaches, however suffer significant disadvantages. First, the analysis of cells in bulk lacks the information on the intercellular variability or the presence of distinct cell subpopulations. Furthermore, cell synchronization causes growth imbalance [60] and leads to altered (“unscheduled”) expression of cyclins [61]. It also triggers intense DNA damage signaling, the indicator of a replication stress [62]. The results obtained by this approach therefore, unlike the presently reviewed methods, have a certain degree of bias and may not precisely reflect the status of the cells otherwise progressing unperturbed through the cycle.

Reviewed are the proteins cyclin D1, p21, Cdt1 and p12 that are being degraded prior to initiation of DNA replication (Figure 1). The pattern of their expression in G_1_ vis-à-vis initiation of DNA replication provides clues on the “time window” between these events and thus on the sequence of their degradation with respect to each. As is evident, there were 75%, 80%, 46%, and 33%, of the D1-, p21-, Cdt1-, or p12- negative cells in G_1_ that did not initiate DNA replication, respectively. These data thus indicate that degradation of cyclin D1 and p21 preceded that of Cdt1, and that p12 was degraded last. Such sequence of the events was reproduced in the repeated experiments [20]. This succession of protein degradation suggests that the sequential removal of cyclin D1, p21 and Cdt1 are the preparatory steps to the formation of Pol δ and the loss of p12 appears to directly precede DNA replication, consistent with prior findings [20, 41-46]. With regard to the temporal regulation of the degradation of the CRL4^Cdt2^ substrates, this can be regarded at the biochemical level as dictated by the affinities of their respective degrons for PCNA, the platform on which they are recognized and degraded, as well as their relative abundances in the cell [20]. However, such approaches do not provide information on the actual kinetics in the cellular mileau. The use of EdU labeling and LSC approaches do reveal the temporal sequence with which these substrates are degraded, at least in relation to the initiation of DNA synthesis. This analysis encompasses the earliest stages of DNA synthesis and captures also those cells just beginning to initiate DNA synthesis that formally by their DNA content are still identified as in G_1_. Thus, these approaches may be of some utility as a means of studying temporal events in the complex process of the initiation of DNA replication.

Interesting also is comparison of the correlation between termination of DNA replication and reappearance of these proteins in G_2_ phase of the cell cycle. This can be estimated from the frequency of the cells with a G_2_M DNA content that are still replicating DNA (entering G_2_M during duration of the EdU pulse) while showing absence of the respective protein. These cells provide the estimate of a length of time between the completion of EdU incorporation and accumulation of the protein. As is seen in Figure 1, the lowest percentage of cells with a G_2_M DNA content that are EdU positive and negative with respect to the respective protein is in the case of p12 (29%), which is distinctly lower than for cyclin D1 (51%), p21 (57) or Cdt1 (55%). Termination of DNA replication, thus, is being initially followed by reappearance of p12 and later by Cdt1, Cyclin D1 and p21.

It can be argued that the rate of EdU incorporation in individual cells may be influenced by its rate of penetration through plasma membrane as well as by equilibration and competition with the endogenous pool of dT and this may bias the estimate of the time of initiation and the early rate of DNA replication. We have recently observed, however, that EdU penetration through plasma membrane and incorporation into DNA is very rapid, viz. as short as 30 sec duration of A549 cells exposure to EdU results in the detectable level of its incorporation [15]. Given the above, the assessments of initiation and the rate of DNA replication are unlikely to be affected by limitations of the accessibility and conversion of EdU to its triphosphate precursor.

## POSITIVE MARKERS OF DNA REPLICATION: CYCLIN E, CYCLIN A, PCNA AND KI-67

### Cyclin E

As mentioned while discussing cyclin D1, phosphorylation of pRb by the cyclin D-Cdk4/6 releases E2F factors that trigger transcription of several genes, including that of cyclin E [63, 64]. Cyclin E forms a complex with CDK2 and this kinase plays a critical role in cell progression through G_1_ and in the G_1_ to S phase transition. Cyclin E/CDK2 phosphorylates retinoblastoma protein (Rb) to promote G_1_ progression [28, 29, 65]. Hyper-phosphorylated Rb can no longer bind to E2F transcriptional factor, releasing it to promote expression of genes essential to drive cells through G_1_ phase to S. During G_1_ and S cyclin E/CDK2 also phosphorylates p27 and p21 [65]. A key mediator of the TGF-β pathway that inhibits cell cycle progression Smad3 can also be phosphorylated by cyclin E/CDK2 and its phosphorylation which inhibits its transcriptional capability ultimately facilitates cell cycle progression [66]. Likewise, cyclin E/CDK2 phosphorylation of CBP/p300 and E2F-5 induces other transcriptional events that promote cell cycle progression [64]. To facilitate histone genes transcription during cell cycle cyclin E/CDK2 phosphorylates p220(NPAT) [65]. Still another substrate of E/CDK2 is nucleophosmin (NPM) whose phosphorylation leads to its release from binding to an unduplicated centrosome thereby triggering centrosome duplication [66]. Additionally, cyclin E/CDK2 phosphorylates CP110 which promotes centriole duplication and centrosome separation [67].

**Figure 2:**
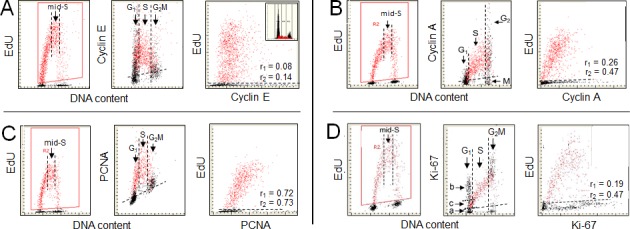
Expression of cyclin E, cyclin A, PCNA and Ki-67 in relation to incorporation of EdU Similar as described in the legend to Fig. 1 A549 cells were exposed to EdU for 60 min and incorporation of EdU was correlated with expression cyclin E, cyclin A, PCNA or Ki-67 and also related to cellular DNA content [11-15, 20]. The cells incorporating EdU were gated (left panels; red dots) and on the bivariate scatterplots correlated with expression of the respective protein, detected immunocytochemically (mid- and right- panels). Additional gating was done to select the mid-S phase cells as shown on the left panels by the parallel vertical lines. The DNA content frequency histogram representing cells from the studied culture is shown in the A right panel. As described in legend to Fig. 1 the dashed skewed lines in mid-panels show the upper level of fluorescence intensity of the cells not incubated with the primary Ab (mid-panels). The correlation coefficients between DNA replication of the mid-S gated cell population (r_1_ = x), or of the all EdU-positive cells (r_2_ = x) and expression of the respective proteins are shown in the right panels. {Figs. 1 and 2 are adapted from ref. [20] with permission of authors and publisher}.

The relationship between expression of cyclin E and incorporation of EdU is shown in Figure 2A. The gating analysis clearly demonstrates that most of the cyclin E negative cells did not initiate DNA replication. This is evident in the middle panel which shows that the cells incorporating EdU, including essentially the cells which were initiating incorporation during the pulse of EdU, were cyclin E positive. The initiation of EdU incorporation thus starts with accumulation of cyclin E to the level that can be immunocytochemically detected. However, a notable number of cells with a G_1_ DNA content and high expression of cyclin E that did not initiated EdU incorporation is evident (Figure 2A, mid-panel). These cells, thus, despite an increase in cyclin E content, are still being held in G_1_ apparently by other factors than by the deficit in amount of this cyclin. The level of cyclin E expression drops during progression of cells through S and even more at the transition to G_2_. Since the cells with G_2_M DNA content are essentially cyclin E negative the stepwise degradation of cyclin E during progression through S appears to be nearly completed at the time of S to G_2_ transition or early in G_2_.

There is a distinct intercellular variability in the amount of incorporated EdU among the cells in the mid-S phase, i.e., the cells that were exposed to the precursor for the full duration (60 min) of the labeling as seen on the scatterplot representing EdU *vs* cyclin E (Figure 2A; left panel). This is also evident in the case of EdU *vs* cyclin A (B) or EdU *vs* PCNA (C). It is likely that this variability is due to different rates of DNA replication in individual cells. Since each of these proteins is associated with the machinery of cell cycle progression it can be expected that its abundance during S phase may be correlated with DNA replication rate as revealed by EdU incorporation. To explore such a possibility the mid-S phase cells were selected by the secondary gating as shown in Figure 2A, 2B, 2C and 2D, left panels. A correlation between incorporation of EdU and the level of expression of these proteins, measured in individual mid-S phase cells, was then analyzed. In the case of cyclin E the regression analysis of this cell subpopulation revealed no correlation between these two variables (r_1_ = 0.08). However, the correlation between EdU and cyclin E level among all EdU-positive cells, which includes in addition to the mid-S phase also the cells undergoing G_1_ to S and S to G_2_ transition during duration of the pulse, was a bit stronger (Figure 2A; r_2_ = 0. 14). Thus, incorporation of EdU appears not to be correlated in any significant way with the abundance of this protein, neither during mid-S nor throughout whole S phase length. This apparent lack of a correlation between cellular content of cyclin E and initiation of DNA replication can be explained that in addition to Rb a multitude of targets that are not directly driving the cell through G_1_S are being phosphorylated by the cyclin E/CDK2 [29, 63-67].

### Cyclin A

Cyclin A regulates progression through the cell cycle at two distinct phases. Its association with CDK2 is required for passage through S phase whereas association with CDK1 drives the cell into mitosis [68-70]. Cyclin A starts to accumulate during S and is abruptly destroyed just prior to metaphase. During the duration of S phase, cyclin A resides in the nucleus where it is involved in the initiation and completion of DNA replication [71, 72]. During initiation, cyclin A/CDK2 phosphorylates several constituents of the DNA replication machinery including CDC6, whose function is of importance for initiation of DNA replication as well as for restriction of the initiation only once per cell cycle. During mitosis cyclin A regulates kinetochore microtubules to promote faithful chromosome segregation [72]. Interestingly, whereas vertebrate cells can enter mitosis in the absence of cyclin A and chromosome segregation is essentially preserved, the incidence of miss-segregation in cells lacking cyclin A is increased [73]. This would indicate a role of cyclin A/CDK1 activity in preservation of faithfulness of chromosome segregation during mitosis [72, 73].

The relationship between expression of cyclin A and incorporation of EdU is presented in Figure 2B. In contrast to expression of cyclin D1 (Figure 1A), it is the presence of cyclin A rather than its absence that is associated with DNA replication. As shown by us previously based on incorporation of BrdU [74, 75] and seen presently, the expression of this protein is confined to cells in late G_1_, S and G_2_. Accordingly, all EdU incorporating are cyclin A positive. Its content progressively increases with cell advancement through the S phase, peaks late in S and in G_2_, and is followed by degradation at the entrance to mitosis, concurrently with phosphorylation of Ser10 of histone H3 in the mitotic cells [76]. The latter, thus can be identified as having a G_2_M DNA content and being cyclin A negative (Figure 2B; mid panel) and phosphorylated H3 positive [76].

As discussed above in reference to cyclin E, the observed intercellular variability in the mid-S phase may be due to differences in rates of DNA replication in individual cells. Because cyclin A is associated with the machinery of the cell progression through S even more so than cyclin E, it is interesting to assess the correlation between cyclin A and EdU incorporation. The regression analysis of the mid-S cell subpopulation revealed relatively weak positive correlation between EdU incorporation and cyclin A expression (r_1_ = 0.26), although somewhat stronger than in the case of cyclin E. However, the correlation between EdU and cyclin A level among all EdU-positive cells, which includes in addition to the S phase also the cells undergoing G_1_ to S and S to G_2_ transition during the duration of the pulse, is distinctly stronger (r_2_ = 0.47). Thus, whereas the presence of cyclin A is closely associated with incorporation of EdU, from its onset at the G_1_ to S transition to the termination at the entrance to G_2_, the abundance of this cyclin appears to be moderately correlated with rate of EdU incorporation.

As mentioned, the correlation between DNA replication and expression of the cyclins D1, E and A is consistent with cytometric analysis of these cyclin proteins based on analysis of BrdU incorporation and SBIP methodology presented in the prior reports [74-76]. The presented data conform also to the assessments of the cell cycle kinetics based on multiparameter analysis of cyclins expression with respect to other proteins of the cell cycle machinery as reported by Jacobberger and his colleagues [77-79]. Their studies however were based on static analysis of presentation of these proteins vis-a-vis cellular DNA content (cell cycle phase) and did not include the kinetic information pertaining initiation and completion of DNA replication, or the rate of DNA replication that can be inferred from the degree of EdU incorporation during the pulse labeling. The presently reviewed data on cyclins are also in accordance with the findings of Furia et al., [16, 17] and complement these earlier cytometric studies by offering a more complete view on a relationship between the intracellular content of the investigated cyclins and DNA replication.

### Proliferating cell nuclear antigen (PCNA)

PCNA was originally recognized as an antigen characteristic of proliferating cells that is expressed in cells nuclei during S phase of the cell cycle [80]. PCNA is a widely recognized cell proliferation marker serving also as a prognostic indicator for variety of tumors [81, 82]. This protein is the DNA sliding clamp that serves as the processivity factor for Pol δ and also as a docking platform where other proteins dock to carry out different processes related to DNA replication and repair [83-87]. The proteins interact with PCNA through the two interacting motifs: PCNA-interacting peptide (PIP) box [88, 91] and AlkB homologue 2 PCNA interacting motif (APIM) [89]. The proteins binding through the PIP-box are primarily associated with DNA replication while these binding through APIM are more important in DNA repair [90].

The relationship between expression of PCNA and DNA replication is presented in Figure 2C. There is a striking similarity between the patterns of EdU incorporation (left panels) *vs*. that of PCNA expression (mid-panel) in relation to DNA content. The gating analysis reveals that all cells that incorporate EdU do also express PCNA and the cells initiating DNA replication have minimal content of this protein. The expression of PCNA as well as incorporation of EdU both peak in mid-S phase. In analogy to expression of cyclin E (Figure 2A) and cyclin A (Figure 2B) the mid-S phase cells incorporating EdU were selected to assess a correlation between expression of this protein and EdU incorporation. The correlation between these variables is significantly stronger for PCNA (r_1_ = 0.72) compared to that assessed for cyclin A or cyclin E. Essentially the same degree of correlation between EdU incorporation and expression of PCNA is seen in the case of all cells replicating DNA i.e. including cells entering- and exiting- S phase during duration of the pulse (r_2_ = 0.73) as for the cells in the mid-S phase.

### Ki-67 protein

The protein Ki-67 is perhaps the most widely used indicator of cycling cells [91]. Although this marker has been known for over three decades [91, 92] its molecular structure and role in cell cycle progression has only recently begun to be elucidated. The reported data indicate that the Ki-67 protein is the key factor in RNA polymerase I dependent nucleolar rRNA synthesis [93-95]. Its expression thus is associated with the production and accumulation of ribosomal RNA. The actuality that Ki-67 is such a dependable marker of proliferation is consistent with results of our early studies showing that the content of cellular rRNA strongly correlates with cell proliferation and its abundance can be used to distinguish the cycling from non-cycling cells [96, 97]. Many subsequent studies have confirmed value of cellular RNA content as a determinant of cells progressing through the cycle [98, 99], also as being correlated with the rate of traverse through the cycle [100, 101], as well as being associated with tumor prognosis [102].

The relationship between DNA replication and expression of Ki-67 protein is shown in Figure 2D. The pattern of Ki-67 expression vis-a-vis DNA replication reveals a striking heterogeneity of the G_1_ cell subpopulation inasmuch as it shows the presence of three subgroups along the Ki-67 coordinate, one the Ki-67 negative (a), the second strongly positive but not initiating DNA replication (b), and the third one, intermediate in expression of Ki-67, from which the cells initiate to incorporate EdU (c). This subdivision of cells in G_1_ phase resembles their classification onto G_1Q_, G_1A_ and G_1B_ compartments based on content of cellular RNA (rRNA) and defined as quiescent (G_1Q_, with minimal RNA content), temporarily noncycling, in the growth phase, accumulating rRNA and protein, prior to the “restriction point” R, (G_1A_, with mid-RNA content), and cells that passed the restriction point, undergoing transition to S (G_1B_, with maximal RNA content) [98, 99]. In analogy to the RNA content as a marker, most likely that: the “a” cells, Ki-67 negative, represent the cells temporarily withdrawn from the cycle (G_1Q_-like cells); the “b”, Ki-67 positive/EdU negative cells, are in the growth phase (equivalent of G_1A_ cells while “c”, the cells entering S phase are analogous to the G_1B_ cells, which after accumulation of the threshold amount of rRNA or growth to the threshold size (protein content) initiate DNA replication [96-99]. Interestingly, the localization of Ki-67 as assessed by LSC is predominantly in nucleoli [20], consistent with the already mentioned function as a ribosome factory. The subdivision of G_1_ to the above compartments also resembles that based on binding of Mcm6 and PCNA, which also are delineating the presence of the restriction point R [103].

There is an evident relationship between the degree of expression of Ki-67 and advancement of cells through S phase, with much more than doubling in content of this protein at the S/G_2_ interphase compared to cells at G_1_/S transition (Figure 2D). This supports the prior observation that the ratio of Ki-67 to DNA content increased three-fold during S and that this protein was partially degraded in G_2_ prior to mitosis [104]. Similar as in the case of other proteins (Figure 2A, 2B, 2C) we have tested whether the intercellular variability in EdU incorporation of the cells in mid-S phase is correlated with Ki-67 expression. The regression analysis of the mid-S cell subpopulation shows rater weak positive correlation between these variables (r_1_ = 0.19). However, as in the case of cyclin A (Figure 2B), the correlation is significantly stronger when assessed for all cells incorporating EdU, i.e. including the cells entering- and exiting- S phase during the EdU pulse (r_2_ = 0.47).

## EXPRESSION OF THE DNA REPLICATION-ASSOCIATED PROTEINS IN RELATION TO EACH OTHER

**Figure 3:**
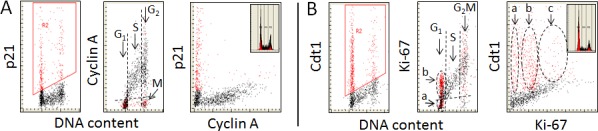
Expression of cyclin A, and Ki-67 in relation the expression of p21^WAF1^ and Cdt1, measured in the same individual cells The individual proteins were detected in A549 cells immunocytochemically using the primary Abs of either mice or goat and the secondary Ab anti-mouse or anti-goat, respectively, labeled with fluorochromes of different emission wavelength; cellular fluorescence was measured by LSC [12-15,20,21]. The gating analysis was based on the selection of cells that were positive in the expression of p21 **A.** or Cdt1 **B.** (left panels; colored red) and the expression of these proteins was juxtaposed with the expression of cyclin A **A.** or Ki-67 **B.**. The insets in the right panels show DNA content frequency histograms from the respective cultures. As described in legend to Fig. 1 the dashed skewed lines in mid-panels show the upper level of fluorescence intensity of the negative control. See the text for further details.

The possibility to concurrently measure different proteins, each tagged with fluorochrome of different color, makes possible to reveal the differential expression of these proteins in individual cells with respect to each other. Figure 3 illustrates the relationship in relative abundance of the positive markers of DNA replication cyclin A, and Ki-67 with respect to p21 and Cdt1, the proteins that are degraded prior to the initiation of DNA replication and synthesized after completion of the replication. In the case of cyclin A (Figure 3A) the data show that essentially all cells expressing p21 (colored red) are cyclin A negative. Of particular interest, however, is analysis of the mitotic cells. As shown by us before [31, 105] mitotic cells are cyclin A negative and therefore, having a G_2_M DNA content, can be identified by cytometry as marked “M” in the mid-panel of Figure 3A. On a close look it is evident they are p21 positive. Thus, the transition from G_2_ to M, while is associated with the loss of cyclin A, is concurrent with the appearance of p21. Because overall rate of protein synthesis is markedly reduced during mitosis [106, 107] it is likely that p21 is synthesized prior to mitosis, in late G_2_. The post-mitotic cells therefore inherit this protein in a significant degree following cytokinesis. The level of p21 is then gradually being reduced during progression through G_1_ so that the cells entering S are p21 negative. These data clearly indicate that there is exclusivity in expression of cyclin A *vs*. p21 in individual cells: when one of these proteins is present the other is absent. This is also exemplified in the right panel of Figure 3A as the total separation of p21 positive- from the cyclin A- positive cells on the bivariate distribution scatterplot.

The bivariate distribution of Ki-67 *vs*. Cdt1 (Figure 3B) resembles to some extent that of Ki-67 *vs*. EdU (Figure 2D). Two subpopulations of Cdt1 positive cells can be identified in G_1_ when displayed with respect to expression of Ki-67 *vs*. DNA content – one having minimal or no expression of Ki-67 (“y”) and the second with elevated expression of this protein (“x”) (Figure 3B, mid-panel). The G_1_ Ki-67 positive cells in all probability correspond to the cells marked “b” plus “c” in Figure 2D, and the S phase cells are, of course, are both EdU and Ki-67 positive. There are numerous Cdt1 negative cells in G_1_ in continuity with the S-phase cells in agreement with the evidence that nearly all Cdt1 negative cells are EdU positive (Figure 1C). Three cell subpopulations can be distinguished on the Ki-67 *vs*. Cdt1 bivariate distribution (Figure 3B; right panel). While the “y” and “x” cells correspond to the same marked on the DNA content *vs*. Ki-67 (mi-panel), the “z” cells represent both the Ki-67 and Cdt1 positive cells in the G_2_ phase of the cell cycle (Figure 2D).

Of interest is to note striking heterogeneity of cells in G_2_M in terms of expression of Ki-67, with some cells being Ki-67 negative while other having the exceptionally high expression of this protein. Also evident is the presence of the Cdt1 positive cells in the G_2_M. Their distribution is likewise very wide (Figure 3B, mid panel) extending from the lowest to the highest level of expression of Ki-67. It is somewhat puzzling to see the presence of Cdt1 positive cells in G_2_M since it has been reported that this protein is being degraded in G_2_ to prevent the re-replication [108, 109]. However, it has been also reported that during rapid cell proliferation Cdt1 can be synthesized shortly after DNA replication, essentially by-passing the G_2_ interval with no induction of re-replication [110]. Furthermore, the presence of Cdt1 alone was reported to be inept to overcome the inhibitory controls that prevent DNA replication in G_2_ as it requires a concurrent presence of Cdc18 to abolish these controls to enable the re-replication [111, 112].

## DNA REPLICATION ASSOCIATED PROTEINS AS MARKERS OF CELL PROLIFERATION IN CYTOPATHOLOGY

PCNA and Ki-67 are well recognized indicators of cell proliferation, frequently assessed in cytopathology as diagnostic and prognostic markers in a variety of diseases, primarily in cancer [113-115]. Expression of cyclin A has also been recently noticed as a proliferation marker and used in clinical pathology [116-119]. Likewise, cyclin E become also recognized in this category of the markers [120-122]. The data presently reviewed reveal a direct relationship between these markers and DNA replication, correlating their expression vis-a-vis incorporation of EdU and also relating to the cell cycle phase as estimated by DNA content. Whereas nearly all cells incorporating EdU are PCNA-, cyclin A-, and cyclin E- positive, of each of these proteins the expression of PCNA (Figure 1) most closely represents the population of cells actually replicating DNA. In the case of cyclin E, its highest levels of expression are in the cells just initiating DNA replication, while PCNA and cyclin A are expressed maximally in the mid-S phase cells. These data thus indicate that immunocytochemical identification of the PCNA-, cyclin A-, and cyclin E- positive cells in pathological specimens provides direct information on DNA replicating cells, equivalent to that otherwise obtained by analysis of incorporation of the DNA precursor. It should be noted however, that the expression of cyclin E (also of cyclin D1) in some tumors, as well as in certain cell lines, was found to be unscheduled [123-125] and thus not exactly as related to DNA replication as presently shown. However because the presence of cyclin A and of PCNA appears to be most strongly associated with DNA replication machinery [30,126-128] and there is no publishable evidence of their unscheduled expression, these proteins appear to be the most faithful indicators of this cellular event.

The relationship between expression of protein detected by Ki-67 Ab and DNA replication is more complex (Figure 2D). This protein, in addition to be present in all DNA replicating cells is also detected in G_1_ and G_2_/M population of cells that show no evidence of EdU incorporation. As discussed earlier in the text, in addition to the DNA replicating cells Ki-67 Ab detects also the cells appear to be in the growth – pre-replicative phase, but it is absent in the cells that are off the cell cycle – (G_0_, G_1Q_ cells) [96-99]. The Ki-67 Ab therefore, has a wider spectrum of detection of the proliferating cells compared with the expression of PCNA, cyclin A or cyclin E, as the presence of the latter proteins is more inclusive to DNA replicating cells.

The distinct absence of expression of cyclin D1, p21, p12 and Cdt1 proteins in the cells incorporating EdU provides the negative markers of DNA replication. Of note, however, is the observation that induction of DNA damage, likely followed by DNA repair, may also lead to the degradation of p12 [45, 46]. If this occurs in G_1_ or G_2_M cells the absence of p12 in such a case not necessarily is an indicator of the S-phase. Detection of the characteristic features of individual cells associated with DNA replication by multiparameter cytometry in addition to offering insights into molecular mechanisms controlling advancement through the cell cycle provides also indicators of cancer progression and stage that have diagnostic and prognostic clinical value.

A note of caution should be added that the immunocytochemical detection of intracellular proteins has shortcomings to be taken into an account when analyzing the cytometric data. The affinity and specificity of different batches of Abs towards epitope of the detected protein can vary, especially in the case of the polyclonal Abs [129]. The problem can be exacerbated by a possibility of cross-reactivity of the Ab with other proteins [130] as well as of auto-fluorescence of cellular constituents [131]. Therefore the detection of the background level of fluorescence to identify the specifically labeled cells based on the use of either the isotype control or the cells treated with the secondary Ab labeled with the fluorochrome only is not always definite. Furthermore, the steric hindrance [132, 133] and mode of cell fixation play a crucial role in the ability of detection of the particular protein. For example, depending on the mode of cell fixation PCNA can be either present only in nuclei of S-phase cells or can also be detected in G_1_ and G_2_ (but not G_0_) phase cells [134-136]. In the first case it is the chromatin/DNA-bound PCNA that after extraction of the detergent-soluble proteins prior to fixation and immuno-staining remains solely in the nucleus of S-phase cells [135, 136]. Otherwise, PCNA is present in G_1_ and G_2_ cells as shown in Figure 2C [20]. The choice of the fixative, cross-linking (e.g. formaldehyde) *vs*. precipitating (e.g. alcohols) plays critical role on detection of different epitopes. All immunocytochemical evidence, therefore, should be critically analyzed with mind of these possible impediments. Supplementation of the immunocytochemical evidence by the Western blotting, e.g. as it was shown in analysis of the p12 protein [45, 53, 54] provides more definite evidence of the presence and relative content of the particular protein in the cell.
